# Optimizing Hydroalcoholic Extraction of African Medicinal Plants for Enhanced α-Amylase Inhibition and Functional Enrichment of Hypoglycemic Bread Doughs

**DOI:** 10.3390/foods15040625

**Published:** 2026-02-09

**Authors:** Mohamed Lemine Lella, Fatma Kallel, Nouha Ben Khaled, Mohamed Vall Ould El Kebir, Mohamed Neifar

**Affiliations:** 1 Food, Nutrition and Metabolic Disorders Unit, Faculty of Science and Technology, University of Nouakchott, King Faiçal Avenue, Nouakchott 2373, Mauritania; mvkebir@hotmail.com; 2 Laboratory of Plant Improvement and Valorization of Agricultural Resources (LAPVA-LR16ES20), National School of Engineers of Sfax (ENIS), University of Sfax, P.O. Box 1173, Sfax 3038, Tunisia; f111fatma@yahoo.fr (F.K.);; 3 Common Services Unit “Bioreactor Coupled with an Ultrafilter”, National School of Engineers of Sfax (ENIS), University of Sfax, P.O. Box 1173, Sfax 3038, Tunisia

**Keywords:** α-amylase inhibition, African medicinal plants, hydroalcoholic extraction optimization, functional bread formulation, antioxidant activities

## Abstract

Type 2 diabetes mellitus (T2DM) remains a global health challenge, necessitating novel therapeutic and dietary strategies. This study optimized hydroalcoholic extraction parameters to maximize α-amylase inhibitory activity from five African medicinal plants: *Combretum glutinosum* (CG), *Ziziphus mauritiana* (ZM), *Gymnosporia senegalensis* (GS), *Boscia senegalensis* (BS), and *Citrullus colocynthis* (CC). A central composite design (CCD) modeled the effects of the liquid-to-solid (L/S) ratio (5–15 mL/g) and ethanol concentration (0–100%, *v*/*v*), identifying optimal conditions at low L/S ratios (5 mL/g) and moderate-to-high ethanol concentrations (40–100%) for GS, ZM, and CG, where inhibition levels exceeded 80–98% of α-amylase activity. Extracts from CG, ZM, and GS showed the strongest inhibition (IC_50_ values of 3.67, 9.8, and 2.25 mg/mL, respectively). Antioxidant capacities, evaluated by DPPH and FRAP assays, correlated strongly with total phenolic content (TPC), with ZM exhibiting superior DPPH (IC_50_ = 1.94 ± 0.16 mg/mL) and FRAP (IC_50_ = 4.34 ± 0.52 mg/mL) activities. Incorporation of optimized plant powders (3%, *w*/*v*) into bread dough significantly influenced textural and colorimetric properties. Mixture design analysis revealed that CG-rich formulations (>2%) yielding hardness exceed 6 N, while ZM–GS blends maintain 3 N, offering targeted firmness control. The addition of medicinal plants significantly increased the total phenolics content by 60% of doughs and thus caused a significant improvement in antioxidant activities. These functional enrichments suggest potential for developing hypoglycemic bakery products with improved sensory attributes. This integrative approach combining extraction optimization and food formulation offers promising avenues for natural antidiabetic agents and functional food development.

## 1. Practical Applications

This study’s findings have practical implications for the food industry, particularly in the design and production of functional bakery products targeting glycemic control. The optimized hydroalcoholic extracts and plant powder blends can be scaled up for industrial extraction and incorporation into staple foods, enabling the development of innovative hypoglycemic bakery items. Food process engineers can apply the optimized extraction parameters and mixture formulations to enhance product quality and functionality while maintaining consumer-acceptable sensory properties. Furthermore, these natural plant-based additives offer a sustainable and culturally relevant approach to managing T2DM through diet, supporting the integration of traditional medicinal plants into modern food processing and product innovation.

## 2. Introduction

Type 2 diabetes mellitus (T2DM) is a chronic metabolic disorder marked by insulin resistance and pancreatic β-cell dysfunction, which leads to sustained hyperglycemia. Over 537 million adults were affected in 2021, with estimates projecting 783 million by 2045, creating heavy demands on global healthcare [1,2]. Current pharmacotherapies, including α-glucosidase and α-amylase inhibitors, effectively manage postprandial hyperglycemia but often cause adverse effects such as gastrointestinal discomfort, leading to increased interest in identifying natural, safer alternatives with multifunctional benefits [3].

Traditional medicinal plants contain bioactive polyphenols, flavonoids, and tannins that target α-amylase and α-glucosidase, the main enzymes in carbohydrate breakdown. By slowing glucose uptake, these compounds blunt postprandial spikes and support better insulin response [3,4]. Their antioxidant effects further address oxidative damage tied to diabetes progression [5,6,7]. African flora, rich in diverse phytochemicals, remains underexplored despite its ethnopharmacological significance [8,9]. This study investigates the medicinal potential of selected Sahelian plants native to Africa’s arid and semi-arid regions—*Combretum glutinosum*, *Ziziphus mauritiana*, *Gymnosporia senegalensis*, *Boscia senegalensis*, and *Citrullus colocynthis*—traditionally harnessed for bioactive compounds that treat ailments such as gastrointestinal disorders, diabetes, inflammation, and infections [10]. Notably, *Combretum glutinosum* is widely used in West Africa against diabetes, with its flavonoid-rich leaf extracts demonstrating hypoglycemic effects in rats, primarily linked to isolated flavonic compounds [11]. *Ziziphus mauritiana* and *Gymnosporia senegalensis* provide nutritional and antidiabetic benefits, while *Boscia senegalensis* offers anti-inflammatory properties, and *Citrullus colocynthis*, a classic antidiabetic cucurbit, exhibits hypoglycemic effects and inhibits carbohydrate-digesting enzymes like α-amylase in rats, as shown in multiple studies [12]. The observation that combining plant extracts or isolated phenolic compounds often yields stronger inhibition of α-amylase/α-glucosidase than any single extract or compound at an equivalent total dose is a phenomenon frequently reported in the scientific literature. α-amylase inhibition plays a crucial role in mitigating postprandial hyperglycemia by delaying the breakdown of complex carbohydrates into oligosaccharides; the inhibition of α-glucosidase and dipeptidyl peptidase-4 (DPP-4) offers complementary mechanisms for enhanced antidiabetic efficacy [13]. The efficacy of plant extracts largely depends on extraction methods and solvent systems, which influence the yield and profile of bioactive compounds [14,15,16]. Hydroalcoholic extraction, combining water and ethanol, is widely employed for its ability to solubilize a broad spectrum of polar and semi-polar phytochemicals [17]. Optimization of extraction parameters such as solvent concentration and liquid-to-solid ratio is crucial to maximize bioactivity while ensuring economic feasibility [15]. Response surface methodology (RSM) and central composite design (CCD) offer robust statistical tools to systematically optimize these variables [14,18,19].

Functional foods enriched with bioactive plant ingredients represent a promising strategy for diabetes management, delivering health benefits beyond basic nutrition [16,17,18]. Among these, plant-derived α-amylase inhibitors are increasingly explored as functional ingredients to improve postprandial glycemic control while also targeting oxidative stress, a major contributor to diabetes complications. Many medicinal plants and herbal mixtures exhibit a dual activity profile, combining α-amylase/α-glucosidase inhibition with strong antioxidant capacity, often mediated by phenolic compounds such as gallic, ellagic, and chlorogenic acids. These compounds have been shown to reduce hyperglycemia and oxidative damage in experimental diabetes models. The therapeutic potential of phenolic compounds in managing type 2 diabetes mellitus is increasingly recognized, primarily through their multifaceted mechanisms of action [20]. Beyond direct radical scavenging, phenolic compounds can also modulate endogenous antioxidant enzyme systems and chelate metal ions that catalyze free radical formation, thus providing comprehensive protection against oxidative stress [20]. The inhibition of α-amylase and α-glucosidase by phenolic compounds plays a critical role in managing postprandial hyperglycemia. They slow down the digestion and absorption of carbohydrates, leading to a more gradual rise in blood glucose levels after meals. This mechanism is analogous to that of pharmaceutical drugs like acarbose but with potentially fewer side effects. However, this correlation between total phenolic content and α-amylase inhibitory activity is not always observed across all studies or plant extracts. This lack of a consistent correlation can be attributed to several factors related to the complexity of plant matrices, the diversity of phenolic structures, and the specificity of enzyme inhibition mechanisms. For instance, Wang et al. [13] illustrate the relationship between various chemical substances and bio-assays indicates that while TPC generally correlates with antioxidant activities like DPPH, ABTS, and FRAP, its correlation with α-amylase and α-glucosidase inhibition can be less pronounced and more specific to certain compounds.

The thermal stability of these phenolic compounds under high-temperature processing, such as baking, is a crucial consideration for their application in functional foods. While some processing methods can lead to degradation, others might enhance or alter their bioavailability and bioactivity [21,22]. Furthermore, the incorporation of phenolic-rich ingredients into baked products can introduce challenges related to thermal degradation during baking [23,24,25]. Bread, as a globally consumed staple, provides an ideal matrix for incorporating medicinal plant powders due to its versatility and wide acceptance. Incorporation of bioactive plant ingredients into bread offers a promising dietary strategy for diabetes management, with evidence supporting improvements in glycemic control, antioxidant status, and overall metabolic health. However, formulation optimization is essential to balance functional benefits with sensory quality and consumer acceptance [19,20,21]. Several studies have successfully incorporated plant extracts or powders into bakery products to enhance antioxidant capacity and glycemic control [22,23,24]. Numerous plant species, including many used in traditional African medicine, contain bioactive compounds—primarily flavonoids and phenolic acids—with significant α-amylase inhibitory activity, capable of moderating postprandial blood glucose levels [3,25,26]. Despite growing interest in functional bread formulations, research specifically integrating African medicinal plants into bread, particularly with a focus on α-amylase inhibition and comprehensive physicochemical characterization, remains limited. Most studies focus on plant-derived α-amylase inhibitors and their antidiabetic potential, but few directly examine their application in bread matrices [3,22,25,26,27].

This study aims to optimize hydroalcoholic extraction of bioactive compounds from five Mauritanian medicinal plants to maximize α-amylase inhibition using RSM. The antioxidant and phenolic profiles of optimized extracts are evaluated. Furthermore, the functional incorporation of selected plant powders into bread dough is investigated, focusing on texture and colorimetric properties using mixture design methodology. To our knowledge, this is the first study to link optimized extraction for α-amylase inhibition with the development of functional breads from Mauritanian medicinal plants. This integrative approach bridges phytochemical optimization and food formulation, offering novel insights into developing hypoglycemic bakery products from African medicinal plants.

## 3. Material and Methods

### 3.1. Plant Materials

The African medicinal plants *Combretum glutinosum* (CG), *Gymnosporia senegalensis* (GS), and *Ziziphus mauritiana* (ZM) were collected from wild natural habitats in Mauritania during the dry season. Leaves were used for all species. Plant materials were washed, dried, and then ground and sieved prior to physicochemical analyses and cake formulation. Botanical identification was performed and confirmed by Dr. Soula (University of Nouakchott, Mauritania). Voucher specimens were deposited under codes HNM 2406 (*Combretum glutinosum* Perr. ex-DC., Combretaceae), HNM 2410 (*Gymnosporia senegalensis* Loes., Celastraceae), and HNM 1546 (*Ziziphus mauritiana* Lam., Rhamnaceae).

### 3.2. Preparation of Hydroalcoholic Extracts

The plant samples were thoroughly washed with water to remove any dust and impurities, then shade-dried for one week at room temperature to best preserve the volatile phytochemical compounds. After drying, the plant materials were finely ground using an electric grinder and stored in airtight containers at 4 °C to maintain the integrity of the bioactive compounds before extraction [28]. The extracts from the plant materials were prepared by a hydroalcoholic extraction process using a mixture of ethanol and water at different ratios. For each sample, 500 g of crude powder was homogenized with 1000 mL of solvent using an Ultra-turrax T25 homogenizer for 30 min. After extraction, the mixture was filtered through a 180-mesh sieve. The hydroalcoholic extract solutions were then concentrated using a continuous vacuum evaporator and dried under vacuum. The resulting powders were stored at 4 °C until use. The extraction yield was calculated using the following formula:

(1) $$Extraction\;yield\;(\%)=\frac{Mass\;of\;extract\;(g)}{Mass\;of\;drymatter\;(g)}\times 100$$

### 3.3. Pancreatic α-Amylase Inhibition Assay

The inhibitory effect of the plant extracts on porcine pancreatic α-amylase was measured using the dinitrosalicylic acid (DNS) method based on [29]. The extract (100 μL) was mixed with 250 μL of enzyme solution (0.5 mg/mL in sodium phosphate buffer, pH 6.9) and incubated at 37 °C for 30 min. Then, 250 μL of starch solution (1%, pH 6.9) was added and incubated again at 37 °C for 30 min. The reaction was stopped by adding 500 μL of DNS reagent, followed by heating in a boiling water bath for 5 min and cooling to room temperature. After dilution with water, the absorbance was read at 540 nm. Controls without enzyme and a positive control (acarbose) were used to correct background absorbance. α-Amylase inhibition (%) was calculated by comparing the absorbance of control and sample using the following formula:

(2) $$\alpha -Amylase\;inhibition\;(\%)=\frac{Ac-As}{Ac}\times 100$$

where Ac is the absorbance of control and As is the absorbance of the sample. The IC_50_ values of the crude extract, solvent fractions, and acarbose were calculated from the dose-response curve through interpolation from the linear regression analysis.

### 3.4. Experimental Design for Extraction Optimization

Central composite designs (CCDs) have been widely and successfully used to optimize extraction and process parameters in various studies. In this study, a two-factor CCD was employed to optimize hydroalcoholic extraction conditions aimed at maximizing α-amylase inhibitory activity. The independent variables were liquid-to-solid ratio (L/S, 5–15 mL/g) and ethanol solvent concentration (ES, 0–100%, *v*/*v*). Fifteen experimental runs, including factorial, axial, and center points, were performed in randomized order to ensure statistical rigor [30]. The response variable was the percentage inhibition of α-amylase activity by the extracts, modeled using a second-order polynomial:

Y = b_0_ + b_1_ (L/S) + b_2_ (ES) + b_11_ (L/S)^2^ + b_22_ (ES)^2^ + b_12_ (L/S × ES) (3)

where Y is the predicted α-amylase inhibition (%) by extracts of *Gymnosporia senegalensis* (GS), *Ziziphus mauritiana* (ZM), *Combretum glutinosum* (CG), *Boscia senegalensis* (BS), or *Citrullus colocynthis* (CC). L/S and ES represent the liquid-to-solid ratio and ethanol concentration, respectively. b_i_ and b_ij_ are the regression coefficients for linear, quadratic, and interaction effects.

### 3.5. Antioxidant Activity

#### 3.5.1. DPPH Free Radical Scavenging Activity

The antioxidant capacity using the DPPH (2,2-diphenyl-1-picrylhydrazyl) radical assay was evaluated as described by Huamán-Castilla et al. [31]. A solution of 0.1 mM DPPH in methanol was prepared, and hydroalcoholic extracts were also dissolved in methanol. Briefly, 0.1 mL of extract (serial dilutions 0.1–2 mg/mL in methanol) was mixed with 3.9 mL of DPPH methanolic solution, vortexed, and incubated in the dark at room temperature for 30 min. Absorbance was then measured at 517 nm using a Shimadzu UV–VIS scanning spectrophotometer (UV-mini-1240; Shimadzu, Kyoto, Japan). Methanol (100% scavenging) and methanolic DPPH solution (0% scavenging) served as positive and negative controls, respectively. Results are given as IC_50_ values, indicating the concentration needed to scavenge 50% of DPPH radicals.

(4) $$DPPH\;radical\;scavenging\;activity\;(\%)=\frac{Ac-As}{Ac}\times 100$$

where Ac is the absorbance of DPPH control and As is the absorbance of the sample. The IC_50_ values of the crude extract, and solvent fractions, were calculated from the dose-response curve through interpolation from the linear regression analysis.

#### 3.5.2. Ferric Reducing Antioxidant Power (FRAP) Assay

The FRAP assay was measured as described by Béjaoui et al. [29]. The FRAP reagent is a solution composed of 25 mL of 0.3 mM acetate buffer (pH 3.6), 2.5 mL of 10 mM 2,4,6-Tris(2-pyridyl)-s-triazine (TPTZ) dissolved in 40 mM hydrochloric acid, and 2.5 mL of 20 mM iron (III) chloride. Absorbance of 1500 µL FRAP reagent was measured before (Ac) and 30 min after the addition of 50 µL extract at various concentrations (0.1–5 mg/mL) at 593 nm using a Shimadzu UV–VIS scanning spectrophotometer (UV-mini-1240; Shimadzu, Kyoto, Japan). Trolox (0–1000 µM) was used to create the calibration curve. The ferric reducing antioxidant potential was expressed as µmol Trolox equivalents (TE) per mL of extract.

(5) $$FRAP\;assay\;(\%)=\frac{Ac-As}{Ac}\times 100$$

where Ac is the absorbance of FRAP reagent control and As is the absorbance of the sample after 30 min. The IC_50_ values of the crude extract, and solvent fractions, were calculated from the dose-response curve through interpolation from the linear regression analysis.

### 3.6. Total Polyphenol Quantification

The total phenol content of the GH extracts was determined by using Folin–Ciocalteu reagent following the method of Huamán-Castilla et al. [31]. Briefly, 1 mL of sample was mixed with 1 mL of Folin–Ciocalteu’s reagent for 3 min, 1 mL of saturated sodium carbonate (Na_2_CO_3_) solution was added to the mixture, and 10 mL distilled water was then adjusted. After 1 h of incubation in the dark at room temperature, the absorbance at 750 nm was measured. The total phenolic contents were determined from the linear equation of a standard curve prepared with gallic acid. The results were expressed as milligrams of gallic acid equivalents per gram of extract (mg GAE/g).

### 3.7. Bread Dough Preparation and Textural and Colorimetric Analyses

Bread doughs were prepared by mixing 250 g of wheat flour (type unspecified) with 3% (*w*/*w*, flour basis) medicinal plant powders (ZM, GS, or CG) using an Alveograph MA82. This fortification level was selected based on prior dose-response experiments, which demonstrated an optimal balance between enhanced bioactivity and preservation of dough quality. Dough water content was carefully adjusted and kept constant for all batches to ensure uniform hydration, while mixing times were strictly controlled to achieve consistent dough development. The applicability of wheat flour for breadmaking was validated by measuring its alveographic properties including maximum overpressure (P), indicating the dough’s resistance to extension; average bubble rupture distance (L), reflecting dough extensibility; and deformation energy (W), representing overall dough strength [32].

The texture properties of dough samples were evaluated using a Texture Analyser (Lloyd Instruments, Leicester, UK). Cylindrical dough discs, 25 mm in diameter and 20 mm in thickness, were prepared with a circular shape cutter from the dough remaining at 25 °C. The analysis involved double compression of the samples within their original containers. Data acquisition was performed with dedicated software from Texture Technologies Corp. (Scarsdale, NY, USA). From the force–time curves, the following parameters were determined: hardness, cohesiveness, gumminess, springiness, and adhesiveness [32].

The colorimetric properties of the dough samples were measured using a colorimeter (Minolta CR-300, Osaka, Japan) to quantify their color on the Hunter Lab scale (L*, a*, b*), with results expressed as lightness, redness/greenness, and yellowness/blueness [33].

### 3.8. Mixture Design for Dough Formulation Optimization

Mixture design is a well-established statistical approach used to optimize dough quality by systematically assessing how varying ingredient proportions influence its properties [34]. In this study, a three-component mixture design was applied to optimize the textural and colorimetric characteristics of wheat dough fortified with *Ziziphus mauritiana* (ZM), *Gymnosporia senegalensis* (GS), and *Combretum glutinosum* (CG) powders at a total concentration of 3% (*w*/*w*). The design comprised seven formulations: the three pure components (100% of each powder), three binary blends at equal ratios (50:50), and one ternary blend with equal proportions (33.3% each). Each formulation was prepared and analyzed in duplicate to account for experimental variability. Key response variables measured included dough textural attributes and colorimetric parameters. The data were modeled using the following polynomial mixture model:

(6) $$Y\;=\;b_{1}\;ZM\;+\;b_{2}\;GS\;+\;b_{3}\;CG\;+\;b_{12}\;\mathrm{ZM-GS}\;+\;b_{13}\;\mathrm{ZM-CG}\;+\;b_{23}\;\mathrm{GS-CG}$$

where Y represents the response variables—Y_1_, hardness (N); Y_2_, cohesion; Y_3_, elasticity (mm); Y_4_, masticability (N); Y_5_, lightness L^*^; Y_6_, redness/greenness a^*^; and Y_7_, yellowness/blueness b^*^. ZM, GS, and CG denote the proportions of *Ziziphus mauritiana, Gymnosporia senegalensis*, and *Combretum glutinosum* powders, respectively. bi and bij are the regression coefficients corresponding to linear and interaction effects.

### 3.9. Statistical Analysis

All experimental data were expressed as mean ± standard deviation based on triplicate measurements to ensure reliability and reproducibility. Statistical significance among groups was evaluated using one-way analysis of variance (ANOVA) followed by Tukey’s post hoc test, with significance considered at *p* < 0.05. For optimization studies, regression models were validated through ANOVA, examining the model significance (F-test), coefficient of determination (R^2^), adjusted R^2^, and lack-of-fit tests to assess goodness of fit and predictive accuracy. Data modeling and graphical analyses, including response surface plotting, were conducted using NemrodW software (Version 9901), facilitating visualization of interaction effects and optimal formulation conditions [30].

## 4. Results and Discussion

### 4.1. Extraction Optimization, Yields, and Bioactivity Correlations

The central composite design (CCD) was applied to model and visualize the combined effects of the liquid-to-solid ratio and ethanol concentration on the percentage inhibition of α-amylase by the five plant powders (GS, ZM, CG, BS, and CC). As shown in Table 1, the experimental and calculated inhibition values are very similar for all plants across the investigated domain (5–15 mL/g, L/S; 0–100% *v*/*v*, ethanol concentration), indicating that the fitted second-order equations reliably predict α-amylase inhibition. GS and ZM exhibited the highest inhibition levels, reaching 92–98% at low L/S ratios and high ethanol concentrations, whereas BS and especially CC consistently showed much lower inhibition (<15–40% in most runs), revealing marked interspecies differences in anti-amylase potential. At the design center (10 L/S, 50% ethanol; runs 9–12), the moderate dispersion of replicate values around the mean provided a sound estimate of pure error and demonstrated good experimental reproducibility.

The ANOVA of the CCD models (Table 2) indicates that for all responses, regression effects are statistically significant, with F-ratios between about 7.8 and 18.9 and *p*-values below 0.05 or 0.01, meaning that the quadratic models explain a large proportion of the variability in α-amylase inhibition as a function of L/S ratio and ethanol concentration. In contrast, lack of fit is non-significant for every extract, indicating that the residual discrepancies between observed and predicted responses are not greater than the inherent experimental error, so the models can be considered adequate within the explored design space. The relatively small residual mean squares compared with the regression mean squares, together with R^2^ values exceeding 0.85, further attest to the robustness and predictive power of the models and justify the use of response-surface plots for interpreting and optimizing the extraction conditions. ANOVA-guided optimization precisely ranks antidiabetic potential: ZM/GS >> CG > BS/CC.

Inspection of the model coefficients (Table 3) clarifies how each factor acts on the response. For ZM (Y2) and CG (Y3), the intercepts are highly significant, and both linear terms in L/S and ethanol concentration are significant, with negative coefficients for L/S and positive (ZM) or negative (CG) coefficients for ethanol concentration. This pattern indicates that decreasing the L/S ratio strongly enhances inhibition for both species, whereas the effect of ethanol is plant-dependent: increasing ethanol promotes inhibition in ZM but reduces it in CG. In ZM, all quadratic and interaction terms (L/S^2^, S^2^, and L/S×S) are also significant, revealing a strong interaction between the two factors, so that the influence of ethanol depends on the selected L/S level and vice versa. By contrast, in CG only the linear terms are significant, while quadratic and interaction terms are not, which suggests an essentially linear response surface over the studied range, with limited curvature. For BS (Y_4_) and CC (Y_5_), the intercept and linear terms are significant and negative for both L/S and S, confirming that lower solvent volume and, within the tested range, lower ethanol concentration favor higher inhibition, although absolute activity remains modest. In CC, the significant positive coefficients for S^2^ and for the interaction L/S×S point to a marked curvature and a synergistic effect between the two factors, leading to a well-defined optimum at low L/S and intermediate ethanol concentration despite the generally low inhibition levels. Finally, GS (Y_1_) has a significant intercept and mixed terms (significant linear S and S^2^), yielding a satisfactory fit despite some nonsignificant coefficients.

**Figure 1:**
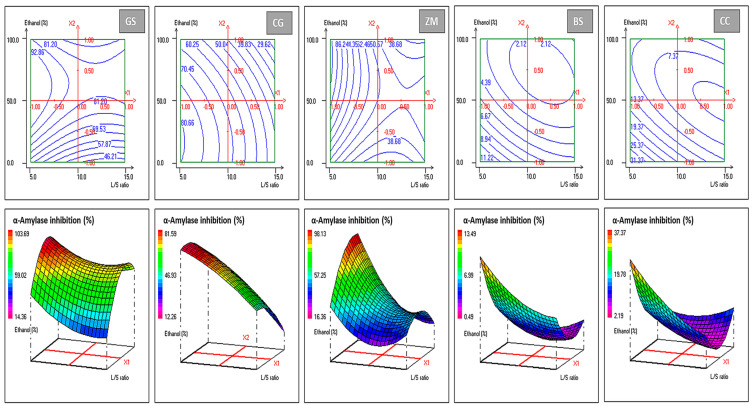
Contour plots illustrating the effect of liquid-to-solid ratio and ethanol percentage on the α-amylase inhibitory activity of the five plant extracts.

The response-surface plots (Figure 1) are fully consistent with these statistical results. For GS, ZM, and CG, the highest α-amylase inhibition is located in the region of low L/S ratio (around 5 mL/g) combined with intermediate to high ethanol concentrations, illustrating that concentrated extracts prepared with limited solvent volume and adequately polar ethanol–water mixtures yield the strongest inhibitory effects. Progressively increasing the L/S ratio or shifting toward very low ethanol contents moves the surfaces toward lower inhibition zones, reflecting dilution of bioactive constituents and sub-optimal solvent polarity. In the case of BS and CC, the surfaces are much flatter and lie at low inhibition values, confirming that these extracts are intrinsically weaker inhibitors. Overall, the response surface patterns (Figure 1) corroborate the ANOVA (Table 2) and coefficient analyses (Table 3), confirming well-defined optima for ZM, GS, and CG at low L/S ratios (~5) and moderate-to-high ethanol concentrations (40–100%), where inhibition routinely exceeds 80%, making these extracts prime candidates for functional bread enrichment. By contrast, CC and BS surfaces remain flat and low (<15% maximum), constraining their efficacy even under optimal conditions.

These response surface patterns and bioactivity profiles align seamlessly with the established literature on botanical α-amylase inhibitor optimization, where low liquid-to-solid (L/S) ratios consistently maximize inhibition through concentrated extracts, while ethanol–water mixtures tuned to polyphenol solubility (typically 40–70%) govern extraction efficiency [31,35]. The observed ranking (ZM/GS >> CG > BS/CC) and divergent ethanol responses mirror phytochemical diversity across species, validating CCD’s utility for plant-specific protocol development [36].

Extraction yields differed markedly among the plants (Table 4), with GS providing the highest recovery (47.90 ± 1.30%), well above ZM (31.31 ± 0.94%) and especially CG (11.51 ± 0.35%). This pattern suggests that GS has a highly soluble matrix rich in extractable constituents, whereas CG is dominated by more recalcitrant, hydrophobic, or polymeric components that resist solubilization under the tested conditions.

ZM exhibited the highest total phenolic content (932.65 ± 30 mg GAE/g) and superior antioxidant activity, as evidenced by the lowest IC_50_ values in both DPPH (1.94 ± 0.16 mg/mL) and FRAP (4.34 ± 0.52 mg/mL) assays, followed closely by CG (925.50 ± 35 mg GAE/g; DPPH: 2.21 ± 0.18 mg/mL; FRAP: 6.10 ± 0.74 mg/mL). In contrast, GS showed significantly lower phenolic content (297.00 ± 11 mg GAE/g) and weaker antioxidant capacity (DPPH: 2.65 ± 0.21 mg/mL; FRAP: 10.50 ± 1.26 mg/mL). These results highlight a strong correlation between antioxidant capacity and total phenolic content, underscoring marked interspecies differences in bioactive compound extraction and free radical scavenging potential. Several studies have reported similar correlations, emphasizing polyphenols as key antioxidant components across diverse plant varieties [37,38].

As shown in Table 4, GS exhibited the strongest α-amylase inhibition (IC_50_ = 2.25 ± 0.23 mg/mL) despite its low polyphenol content, whereas polyphenol-rich CG and ZM showed weaker effects (IC_50_ = 3.67 ± 0.37 mg/mL and 9.80 ± 0.98 mg/mL, respectively). This apparent mismatch highlights that total phenolic content is a global index that does not necessarily predict enzyme inhibition, which depends on the presence, structure, and affinity of specific inhibitory molecules and on potential synergistic/antagonistic interactions within crude extracts. In GS, the high potency may therefore be driven by non-phenolic or weakly phenolic constituents with direct enzyme-targeting capacity (e.g., gymnemic-acid-like/saponin-type and related triterpenoid metabolites), which can bind the enzyme more effectively than bulk phenolics. In contrast, ZM and CG may rely mainly on classic polyphenol–enzyme interactions that are highly dependent on phenolic composition (degree of polymerization and hydroxylation pattern) rather than total quantity, which can explain why polyphenol-rich extracts do not automatically translate into lower IC_50_ values [38,39]. Consistently, our data indicate no significant correlation between phenolic content and α-amylase inhibitory activity consistent with Oyedemi et al. [40] on traditional plants. The absence of a significant correlation between total phenolic content and α-amylase inhibitory activity does not negate the role of phenolic compounds in enzyme inhibition. Instead, it highlights the need for a more detailed analysis focusing on individual phenolic compounds or specific phenolic subclasses, their unique structural features, and their specific mechanisms of action, rather than relying solely on a bulk measure like TPC [13]. The hydro-ethanolic systems may enrich different families of bioactives across species, meaning that the most active inhibitors in GS could be preferentially extracted even when total phenolics remain low. Overall, these results align with the literature on botanical α-amylase inhibitors, where low liquid-to-solid ratios and ethanol–water mixtures (40–70%) optimize polyphenol extraction and enzyme inhibition, yielding concentrated bioactive extracts [29,34]. α-amylase inhibition is an important aspect of antidiabetic activity, but it is not a standalone predictor of overall efficacy. The concurrent inhibition of α-glucosidase provides a more complete control over postprandial glucose levels, and the additional inhibition of DPP-4 offers a distinct pathway to enhance insulin secretion and improve glycemic regulation. The synergistic interactions of diverse phenolic compounds in natural extracts, along with their inherent antioxidant properties, further emphasize the benefits of a multi-target strategy for combating type 2 diabetes effectively [13]. In conclusion, *Gymnosporia senegalensis*, *Ziziphus mauritiana*, and *Combretum glutinosum* could hold potential as functional food ingredients to regulate carbohydrate metabolism and postprandial hyperglycemia.

Although the present study did not assess post-baking bioactivity, the literature shows that high-temperature processing of cereals, pulses, roots, and teas partially reduces native phenolics while sometimes preserving or even enhancing functional properties. In pigmented maize “proja” and black corn bread, 15–33% of total phenolics are lost, yet DPPH, ABTS, ORAC, and radical scavenging activities are often maintained or increased due to the release of bound phenolics and formation of Maillard reaction products [25,39,40]. Similar trends are observed in whole-grain breads, muffins, and pulse-based baked products, where bound phenolic acids decrease but free phenolics and overall antioxidant capacity rise [39,40]. Because α-amylase and α-glucosidase inhibition depends on polyphenol structure, thermal processing that shifts the balance between free and bound phenolics, degrades some flavonoids, or promotes polymerization and Maillard-derived products may alter—but not necessarily reduce—enzyme inhibitory potency. For example, baking of black tea increased low-polarity polyphenols and significantly enhanced in vitro α-amylase and α-glucosidase inhibition [41]. Collectively, these studies suggest that baking often transforms rather than eliminates bioactive compounds, highlighting the importance of ingredient selection and processing conditions in preserving health-promoting properties [39,40,41].

### 4.2. Impact of Medicinal Plant Powders on Bread Dough Texture and Color

The experimental data from the three-factor mixture design, detailed in Table 5, present the formulation compositions of ZM, GS, and CG at a total concentration of 3% (*w*/*w*), alongside their corresponding measured textural and colorimetric responses and the predicted theoretical values from the fitted quadratic model. These results highlight the blends’ influence on dough rheology, with close alignment between observed and predicted responses indicating robust model adequacy (R^2^ > 0.9 for key parameters).

**Figure 2:**
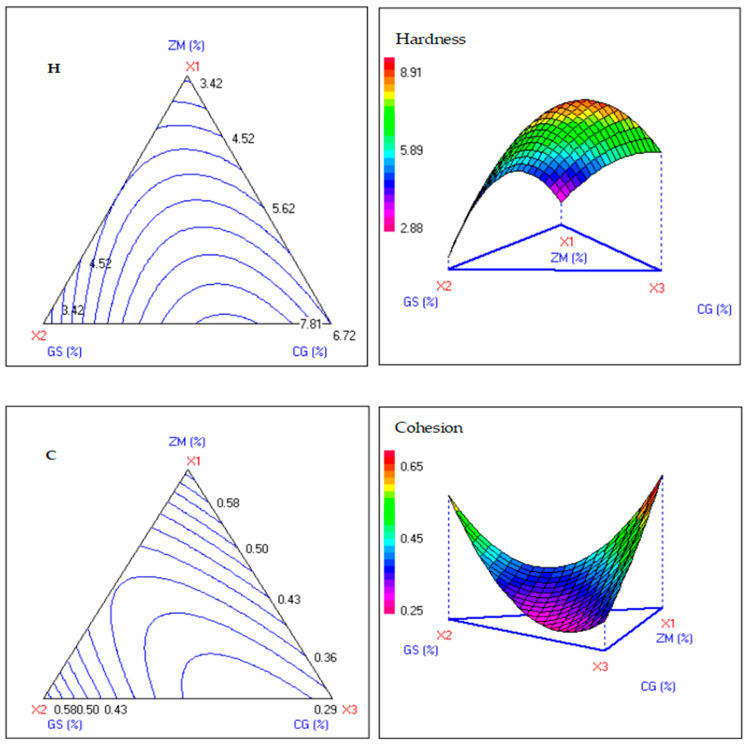

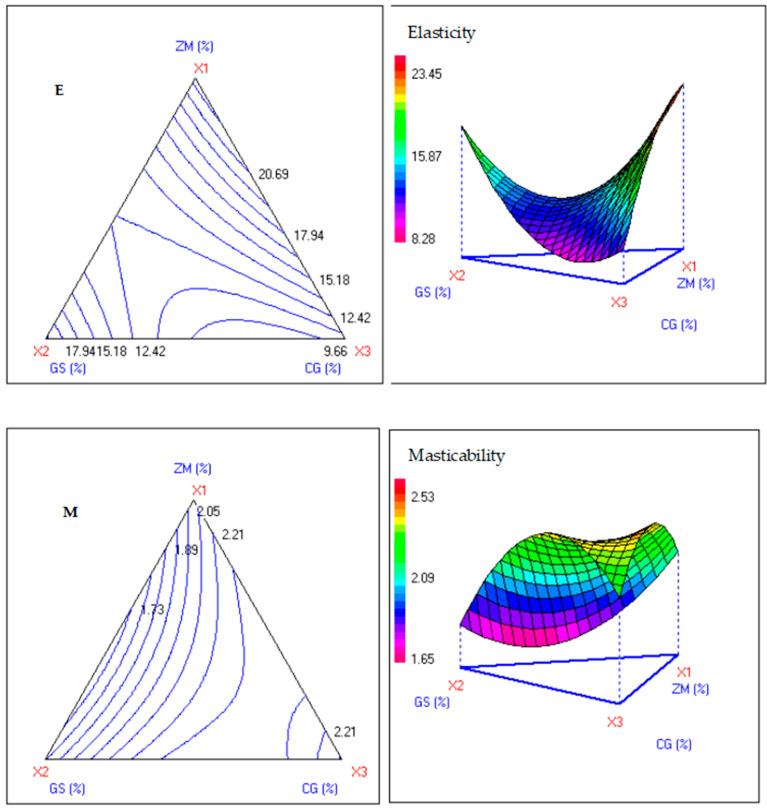
Iso-response plots illustrating the effect of the antidiabetic medicinal plant powders (ZM, CG, and GS) on the bread dough textural properties (H, hardness (N); C, cohesion; E, elasticity (mm); M, masticability (N)).

Hardness (Y_1_) followed a highly significant quadratic model (F = 29.17, *p* < 0.001), with CG exerting the strongest positive linear effect (b_3_ = 6.707, t = 17.01, *p* < 0.001), followed by ZM (b_1_ = 3.281) and GS (b_2_ = 2.935), enhanced by the significant CG–GS interaction (b_23_ = 13.772, *p* < 0.001) (Table 6 and Table 7). This matches the water-binding and gluten-disrupting effects of fiber-rich plant powders, which boost texture profile analysis firmness [42,43]. Iso-response surfaces (Figure 2) confirm CG-rich formulations (>2%) exceed 6 N hardness, while ZM–GS blends maintain 3 N, offering targeted firmness control.

Cohesiveness (Y_2_) showed a significant model (F = 6.66, *p* < 0.05), with positive linear contributions from all components but negative ZM–GS and CG–GS interactions indicating synergistic matrix weakening (Figure 2; Table 6 and Table 7). CG alone reduced cohesiveness from control (0.61) to 0.28–0.36 (exp. 3), while ZM–GS (1.5% + 1.5%) yielded intermediate values (0.34–0.65, exp. 4), reflecting polysaccharides and phenolics modulating water/gluten dynamics, consistent with fiber-protein fragmentation in antioxidant-enriched doughs [44,45,46]. Elasticity (Y_3_) was well fitted by a quadratic model (F = 17.18, *p* < 0.001), where CG reduced elasticity via strong negative interactions with ZM/GS (b_12_ = −34.87, *p* < 0.001; b_23_ = −22.30, *p* < 0.01), while ZM (b_1_ = 22.72) and GS (b_2_ = 19.67) contributed positively; peak elasticity (23.45) occurred with pure ZM (Table 5, Table 6 and Table 7). Contour plots (Figure 2) show optimal ZM–GS mixtures (1–2% each, Y_3_ > 20), where CG strengthens the gluten network, similar to high-fiber wheat doughs [47,48].

Masticability (Y_4_) followed a significant model (F = 6.67, *p* < 0. 05), which was mainly influenced by positive linear effects (ZM: 2.10, GS: 1.80, CG: 2.12; all *p* < 0.05) and CG-GS synergy (b_23_ = 1.534, *p* < 0.05), reaching a maximum of 2.46 for GS-CG (exp. 6) versus control 1.30 (Table 5, Table 6 and Table 7). ZM blends were still preferred (~control levels), whereas CG could be used to enable controlled chewiness increases in fiber-rich breads [49].

**Figure 3:**
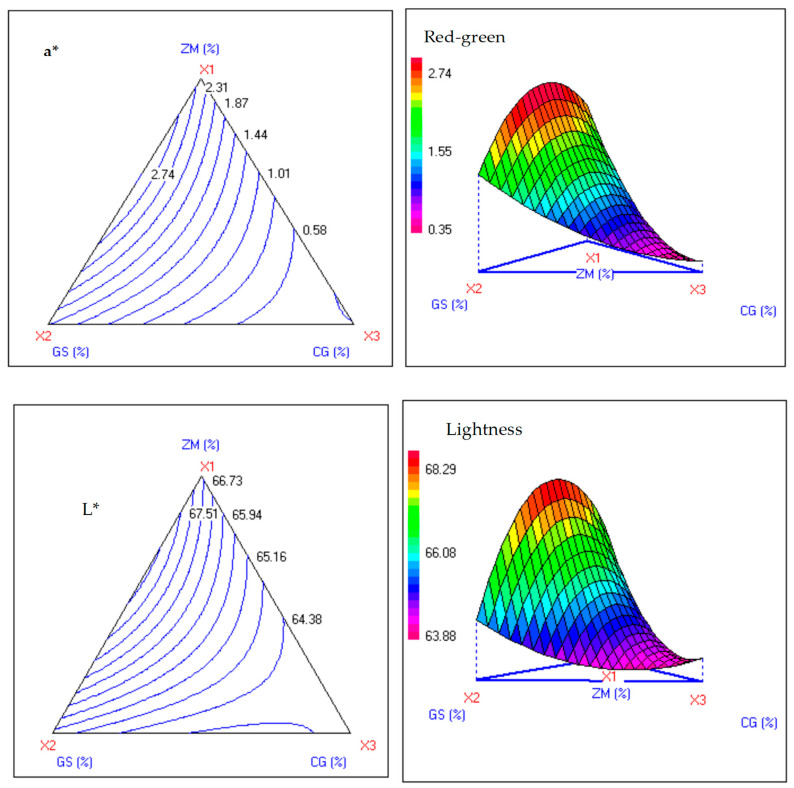

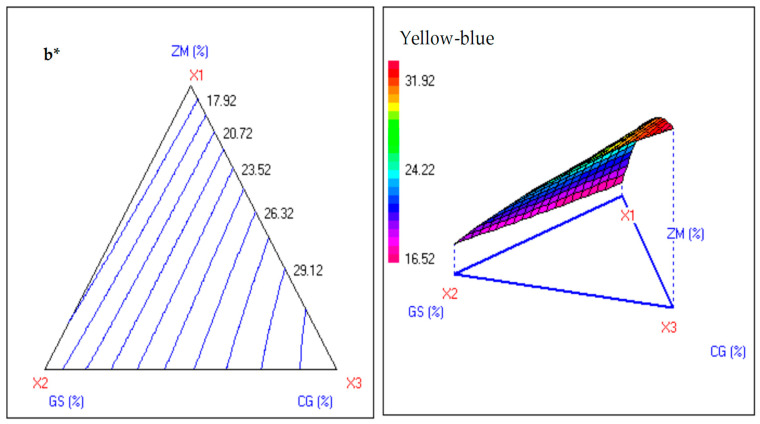
Iso-response plots illustrating the effect of the antidiabetic medicinal plant powders (ZM, CG, and GS) on the bread dough colorimetric properties (lightness (L*), a* (red-green), and b* (yellow-blue)).

Color lightness (L*, Y_5_) followed a quadratic model (F = 13.81, *p* < 0.01). It decreased from control (approximately 80.25) to CG-rich zones (63.99–64.20, exp. 3) through significant linear terms (all *p* < 0.001). Redness (a*, Y6; F = 1850.59, *p* < 0.001) and yellowness (b*, Y_7_; F = 1572.74, *p* < 0.001) increased with CG (a* around 31.4–31.9; b* around 32). This change was enhanced by interactions, resulting in vibrant CG-centered hues compared with lighter ZM–GS (Table 5, Table 6 and Table 7; Figure 3). These differences reflect Maillard and pigment effects in polyphenol-enriched products [35,50]. The ternary design delineates ZM–GS for soft/elastic/light doughs and CG for firm/colored/phytochemical-rich ones, aligning with fiber-phenolic bakery systems [49,51].

Combined with texture responses, the ternary design identifies ZM–GS blends for soft, elastic, light-colored doughs and CG-enriched zones for firmer, strongly colored, phytochemical-rich products, consistent with other plant-enriched bakery systems

The observation that ZM–GS binaries preserve cohesiveness and elasticity while still modifying color is consistent with work on herbal and pomace-enriched breads, where combined low-dose botanicals mitigate individual negative impacts on volume and texture [52,53]. From a nutritional and metabolic perspective, incorporating small amounts of polyphenol- and fiber-rich botanicals is widely used to introduce α-amylase/α-glucosidase inhibitory activity and antioxidant capacity into baked goods, supporting glycemic control without abandoning familiar formats [54,55]. Biscuits and breads fortified with antidiabetic plants or by-products show increased phenolics, stronger radical-scavenging capacity, and significant inhibition of starch-digesting enzymes in vitro, while still achieving acceptable hardness, cohesiveness, and chewiness when properly optimized [54,55]. In addition, compared with the control, the addition of 3% total powder resulted in a significant increase in total polyphenol content by 60% and a parallel enhancement in DPPH radical-scavenging activity. These outcomes mirror the present ZM/GS/CG system, in which 3% total powder delivers measurable textural effects but remains technologically manageable and where ZM-GS-rich formulas appear particularly promising for balancing soft, elastic crumb with antioxidant and antidiabetic bioactivity. While dough rheology and stability provide robust predictions of baked bread quality, as shown in studies linking empirical rheological parameters to bread volume, crumb structure, and texture [56,57], confirmatory baking trials remain essential to fully evaluate product performance. Such trials would allow verification of heat-induced structural transformations, including crumb porosity and gas cell distribution, as well as assessment of the retention of bioactive compounds after baking and consumer-relevant attributes such as crust color and Maillard-derived flavor volatiles. This limitation of the current study is acknowledged and suggested as a perspective for future research.

## 5. Conclusions

In this study, we optimized hydroalcoholic extraction conditions from selected Mauritanian medicinal plants to maximize their α-amylase inhibitory activity, with *Combretum glutinosum*, *Ziziphus mauritiana*, and *Gymnosporia senegalensis* emerging as the most potent, closely associated with their high phenolic content and antioxidant capacity. Incorporation of these optimized powders at 3% (*w*/*w*) into bread doughs resulted in targeted modifications of texture (e.g., controlled firmness) and color (e.g., enhanced redness), demonstrating their potential for hypoglycemic bakery applications. Moreover, supplementation significantly enhanced bread phenolic content and antioxidant activity. Further research should assess phytotoxicity, thermal stability of room-temperature dried powders during baking, and comprehensive baked bread quality—crumb structure, specific volume, crumb firmness, crust color, loaf volume, and bioactivity retention. Concurrently, in vivo glycemic response trials (diabetic rodent models or human volunteers), sensory acceptability panels, gastrointestinal digestibility assays, and matrix expansion (biscuits, cookies, and pasta) will validate industrial viability. Optimizing CG+ZM+GS ternary mixtures for α-amylase/α-glucosidase/DPP-IV synergy could further enhance formulations for palatability, shelf-life stability, and consumer acceptance.

## Figures and Tables

**Table 1:** Experimental conditions of CCD in natural and coded variables and the corresponding experimental and calculated responses.

N° Exp	Factors		Y: Alpha Amylase Inhibition (%) of Different Medicinal Plant Extracts									
	L/S Ratio (mL/g)	S (%, *v*/*v*)	GS (Y_1_)		ZM (Y_2_)		CG (Y_3_)		BS (Y_4_)		CC (Y_5_)	
			Y_exp_	Y_cal_	Y_exp_	Y_cal_	Y_exp_	Y_cal_	Y_exp_	Y_cal_	Y_exp_	Y_cal_
1	5[−1]	0[−1]	74.36	66.16	52.20	54.48	75.66	80.76	13.49	12.49	37.37	33.79
2	15[1]	0[−1]	41.21	36.51	33.56	38.29	42.33	45.96	6.65	5.97	11.45	10.95
3	5[−1]	100[1]	91.69	82.68	98.13	94.12	64.89	61.94	2.79	3.72	13.52	11.51
4	15[1]	100[1]	77.24	76.74	32.73	31.18	24.52	20.09	2.15	3.40	12.26	13.32
5	5[−1]	50[0.0]	90.37	102.57	86.30	88.03	80.66	78.51	5.64	5.69	8.75	14.34
6	15[1]	50[0.0]	77.08	82.27	51.65	48.46	39.39	40.18	2.85	2.27	4.37	3.82
7	10[0.0]	0[−1]	28.71	41.60	35.62	28.60	75.74	67.01	6.04	7.70	15.26	19.34
8	10[0.0]	100[1]	67.99	72.48	39.30	44.86	37.29	44.67	4.22	2.03	8.42	9.38
9	10[0.0]	50[0.0]	92.86	87.69	49.01	50.45	61.54	63.00	3.88	2.45	4.84	6.05
10	10[0.0]	50[0.0]	89.45	82.68	50.06	50.45	63.93	63.00	2.69	2.45	6.31	6.05
11	10[0]	50[0]	82.83	82.68	42.49	50.45	66.59	63.00	0.98	2.45	11.84	6.05
12	10[0]	50[0]	82.98	82.68	58.80	50.45	58.60	63.00	1.75	2.45	6.23	6.05

L/S, liquid to solid ratio; S, solvent (ethanol) concentration; GS: *Gymnosporia senegalensis*; ZM: *Ziziphus mauritiana*; CG: *Combretum glutinosum*; BS: *Boscia senegalensis*; CC: *Citrullus colocynthis*.

**Table 2:** Statistical analysis of variance (ANOVA) for the different CCD models postulated for the five medicinal plants.

Source of Variation	Sum of Squares	Degrees of Freedom	Mean Square	*F*-Ratio	Significance
Y_1_: α-amylase inhibition by GS extract					
Regression	3893.04	5	778.609	7.507	S (*p* ˂ 0.05)
Residuals	617.072	6	102.845		
Lack of fit	543.072	3	181.061	7.351	NS
Pure error	73.887	3	24.692		
Total	4510.12	11			
Y_2_: α-amylase inhibition by ZM extract					
Regression	4317.3	5	863	18.853	S (*p* ˂ 0.01)
Residuals	274	6	45.79		
Lack of fit	139.9	3	46.66	1.0386	NS
Pure error	134.7	7	0.008		
Total	4592	13			
Y_3_: α-amylase inhibition by CG extract					
Regression	3210.49	5	642.099	16.1460	S (*p* ˂ 0.01)
Residuals	238.610	6	39.7684		
Lack of fit	203.814	3	67.9381	5.8575	NS
Pure error	34.795	3	11.5985		
Total	3449.10	11			
Y_4_: α-amylase inhibition by BS extract					
Regression	107.3782	5	21.4756	7.7935	S (*p* ˂ 0.05)
Residuals	16.5335	6	2.7556		
Lack of fit	11.8426	3	3.9475	2.5246	NS
Pure error	4.6909	3	1.5636		
Total	123.9116	11			
Y_5_: α-amylase inhibition by CC extract					
Regression	752.1333	5	150.4267	8.8127	S (*p* ˂ 0.05)
Residuals	102.4156	6	17.0693		
Lack of fit	73.6275	3	24.5425	2.5576	NS
Pure error	28.7881	3	9.5960		
Total	854.549	11			

GS: *Gymnosporia senegalensis*; ZM, *Ziziphus mauritiana*; CG: *Combretum glutinosum*; BS: *Boscia senegalensis*; CC: *Citrullus colocynthis*; S (*p* ˂ 0.01), significant at the level 99%; S (*p* ˂ 0.05), significant at the level 95%; NS, non-significant.

**Table 3:** Estimates of and statistics on the CCD model coefficients.

	Coefficient	F. Inflation	Standard Deviation	t Exp	Significance
GS: Y_1_ = 82.683 − 10.148 L/S + 15.440 S + 9.735 (L/S)^2^ − 25.640 S^2^ + 4.675 L/S. S					
b_0_	82.683		4.629	17.86	S (*p* < 0.001)
b_1_	−10.148	1.00	4.140	−2.45	S (*p* < 0.05)
b_2_	15.440	1.00	4.140	3.73	S (*p* < 0.01)
b_11_	9.735	1.13	6.210	1.57	NS
b_22_	−25.640	1.12	6.210	−4.13	S (*p* < 0.01)
b_12_	4.675	1.00	5.071	0.92	NS
ZM: Y_2_ = 50.455 − 19.782 L/S + 8.130 S + 17.790 (L/S)^2^ − 13.725 S^2^ − 11.690 L/S. S					
b_0_	50.455		3.089	16.33	S (*p* < 0.001)
b_1_	−19.782	1.00	2.763	−7.16	S (*p* < 0.001)
b_2_	8.130	1.00	2.763	2.94	S (*p* < 0.05)
b_11_	17.790	1.13	4.144	4.29	S (*p* < 0.01)
b_22_	−13.725	1.12	4.144	−3.31	S (*p* < 0.05)
b_12_	−11.690	1.00	3.384	−3.45	S (*p* < 0.05)
CG: Y_3_ = 63.003 − 19.162 L/S − 11.172 S − 3.653 (L/S)^2^ − 7.163 S^2^ − 1.760 L/S. S					
b_0_	63.003		2.878	21.89	S (*p* < 0.001)
b_1_	−19.162	1.00	2.575	−7.44	S (*p* < 0.001)
b_2_	−11.172	1.00	2.575	−4.34	S (*p* < 0.01)
b_11_	−3.653	1.13	3.862	−0.95	NS
b_22_	−7.163	1.12	3.862	−1.85	NS
b_12_	−1.760	1.00	3.153	−0.56	NS
BS: Y_4_ = 2.455 − 1.712 L/S − 2.187 S + 1.530 (L/S)^2^ + 2.415 S^2^ + 1.550 L/S. S					
b_0_	2.455		0.758	3.24	S (*p* < 0.05)
b_1_	−1.712	.00	0.678	−2.53	S (*p* < 0.05)
b_2_	−2.837	1.00	0.678	−4.19	S (*p* < 0.01)
b_11_	1.530	1.13	1.017	1.51	NS
b_22_	2.415	1.12	1.017	2.38	NS
b_12_	1.550	1.00	0.830	1.87	NS
CC: Y_5_ = 6.046 − 5.260 L/S − 4.980 S + 3.033 (L/S)^2^ + 8.312 S^2^ + 6.165 L/S. S					
b_0_	6.046		1.886	3.21	S (*p* < 0.05)
b_1_	−5.260	1.00	1.687	−3.12	S (*p* < 0.05)
b_2_	−4.980	1.00	1.687	−2.95	S (*p* < 0.05)
b_11_	3.033	1.13	2.530	1.20	NS
b_22_	8.312	1.12	2.530	3.29	S (*p* < 0.05)
b_12_	6.165	1.00	2.066	2.98	S (*p* < 0.05)

GS: *Gymnosporia senegalensis*; ZM: *Ziziphus mauritiana*; CG: *Combretum glutinosum*; BS: *Boscia senegalensis*; CC: *Citrullus colocynthis*. S (*p* ˂ 0.001): significant at the level 99.9%; S (*p* ˂ 0.01): significant at the level 99%; S (*p* ˂ 0.05): significant at the level 95%; NS: nonsignificant.

**Table 4:** Biological and phytochemical properties of medicinal plant powders.

Extract	Yield (%)	Total Phenols Content (mg GAE/g)	IC_50_ DPPH (mg/mL)	IC_50_ FRAP (mg/mL)	IC_50_ α-Amylase Inhibition (mg/mL)
CG	11.51 ± 0.35	925.50 ± 35	2.21 ± 0.18	6.10 ± 0.74	3.67 ± 0.37
ZM	31.31 ± 0.94	932.65 ± 30	1.94 ± 0.16	4.34 ± 0.52	9.80 ± 0.98
GS	47.90 ± 1.3	297.00 ± 11	2.65 ± 0.21	10.50 ± 1.26	2.25 ± 0.23

GS: *Gymnosporia senegalensis*; ZM: *Ziziphus mauritiana*; CG: *Combretum glutinosum*; FRAP: ferric reducing antioxidant power.

**Table 5:** Three-factor mixing design experimental conditions with measured textural and colorimetric responses.

N° Exp.	ZM (%)	GS (%)	CG (%)	H (N)	C	E (mm)	M (N)	L*	a*	b*
1	3	0	0	3.378	0.624	23.451	2.110	67.23	2.21	16.71
1′	3	0	0	3.205	0.648	22.383	2.078	66.27	2.25	16.52
2	0	3	0	3.016	0.644	20.454	1.943	65.02	1.66	18.20
2′	0	3	0	2.876	0.573	19.279	1.650	64.89	1.68	18.19
3	0	0	3	6.282	0.355	11.507	2.231	64.20	0.36	31.38
3′	0	0	3	7.154	0.278	10.021	1.995	63.99	0.36	31.92
4	1.5	1.5	0	5.076	0.349	11.527	1.775	68.29	2.72	17.39
4′	1.5	1.5	0	4.953	0.338	11.850	1.674	68.03	2.74	17.49
5	1.5	0	1.5	5.511	0.583	20.566	2.217	64.88	0.77	27.17
5′	1.5	0	1.5	5.425	0.282	16.308	2.531	64.66	0.77	27.37
6	0	1.5	1.5	8.909	0.275	9.228	2.457	64.39	0.71	26.13
6′	0	1.5	1.5	7.534	0.250	8.2823	2.284	64.03	0.75	26.34
7	1	1	1	6.283	0.318	12.245	1.995	64.11	1.32	23.64
7′	1	1	1	7.776	0.394	16.199	2.231	65.67	1.39	24.05
Cs	0	0	0	1.92	0.61	22.80	1.30	80.30	1.10	14.10
Cs′	0	0	0	1.90	0.62	21.90	1.30	80.20	1.10	14.10

H, hardness (N); C, cohesion; E, elasticity (mm); M, masticability (N); GS: *Gymnosporia senegalensis*; ZM: *Ziziphus mauritiana;* CG: *Combretum glutinosum*; BS: *Boscia senegalensis*; CC: *Citrullus colocynthis*; Cs: control sample without medicinal plant powder addition.

**Table 6:** Statistical analysis of variance (ANOVA) of different postulated mixture design models of textural and colorimetric responses.

Source of Variation	Sum of Squares	Degrees of Freedom	Mean Square	*F*-Ratio	Significance
Y_1_: Hardness					
Regression	45.6927	5	9.1385	29.1694	S (*p* ˂ 0.001)
Residuals	2.5063	8	0.3133		
Lack of fit	0.0303	1	0.0303	0.0856	NS
Pure error	2.4761	7	0.3537		
Total	48.1990	13			
Y_2_: Cohesiveness					
Regression	0.2499	5	0.0500	6.6647	S (*p* ˂ 0.05)
Residuals	0.0600	8	0.0075		
Lack of fit	0.0057	1	0.0057	0.7290	NS
Pure error	0.0543	7	0.0078		
Total	0.3099	13			
Y_3_: Elasticity					
Regression	322.3321	5	64.4664	17.1842	S (*p* ˂ 0.001)
Residuals	30.0120	8	3.7515		
Lack of fit	10.2656	1	10.2656	3.6391	NS
Pure error	19.7464	7	2.8209		
Total	352.3441	13			
Y_4_: Masticability					
Regression	0.7487	5	0.1497	6.6724	S (*p* ˂ 0.05)
Residuals	0.1795	8	0.0224		
Lack of fit	0.0110	1	0.0110	0.4584	NS
Pure error	0.1685	7	0.0241		
Total	0.9282	13			
Y_5_: L*					
Regression	25.6838	5	5.1368	13.8116	S (*p* ˂ 0.01)
Residuals	2.9753	8	0.3719		
Lack of fit	1.1444	1	1.1444	4.3754	NS
Pure error	1.8309	7	0.2616		
Total	28.6591	13			
Y_6_: a*					
Regression	8.9164	5	1.7833	1850.5889	S (*p* ˂ 0.001)
Residuals	0.0077	8	0.0010		
Lack of fit	0.0033	1	0.0033	5.1266	NS
Pure error	0.0044	7	0.0006		
Total	8.9241	13			
Y_7_: b*					
Regression	397.8814	5	79.5763	1572.7443	S (*p* ˂ 0.001)
Residuals	0.4048	8	0.0506		
Lack of fit	0.1098	1	0.1098	2.6049	NS
Pure error	0.2950	7	0.0421		
Total	398.2861	13			

S (*p* ˂ 0.001): significant at the level 99.9%; S (*p* ˂ 0.01): significant at the level 99%; S (*p* ˂ 0.05): significant at the level 95%; NS: nonsignificant.

**Table 7:** Statistical evaluation of the significance of the mixture design model coefficients estimated for textural and colorimetric responses.

	Coefficient	F. Inflation	Standard Deviation	t Exp	Significance
Y_1_ = 3.281 ZM + 2.935 GS + 6.707 CG + 7.797 ZM.GS + 2.067 ZM.CG + 13.772 CG.GS					
b_1_	3.281	1.60	0.394	8.32	S (*p* ˂ 0.001)
b_2_	2.935	1.60	0.394	7.44	S (*p* ˂ 0.001)
b_3_	6.707	1.60	0.394	17.01	S (*p* ˂ 0.001)
b_12_	7.797	1.57	1.812	4.30	S (*p* ˂ 0.01)
b_13_	2.067	1.57	1.812	1.14	NS
b_23_	13.772	1.57	1.812	7.60	S (*p* ˂ 0.001)
Y_2_ = 0.631 ZM + 0.604 GS + 0.312 CG − 1.022 ZM.GS − 0.082 ZM.CG − 0.707 CG.GS					
b_1_	0.631	1.60	0.061	10.35	S (*p* ˂ 0.001)
b_2_	0.604	1.60	0.061	9.90	S (*p* ˂ 0.001)
b_3_	0.312	1.60	0.061	5.11	S (*p* ˂ 0.01)
b_12_	−1.022	1.57	0.280	−3.65	S (*p* ˂ 0.01)
b_13_	−0.082	1.57	0.280	−0.29	NS
b_23_	−0.707	1.57	0.280	−2.52	S (*p* ˂ 0.05)
Y_3_ = 22.720 ZM + 19.669 GS + 10.567 CG − 34.870 ZM.GS + 10.329 ZM.CG − 22.297 CG.GS					
b_1_	22.720	1.60	1.364	16.65	S (*p* ˂ 0.001)
b_2_	19.669	1.60	1.364	14.42	S (*p* ˂ 0.001)
b_3_	10.567	1.60	1.364	7.74	S (*p* ˂ 0.001)
b_12_	−34.870	1.57	6.272	−5.56	S (*p* ˂ 0.001)
b_13_	10.329	1.57	6.272	1.65	NS
b_23_	−22.297	1.57	6.272	−3.56	S (*p* ˂ 0.01)
Y_4_ = 2.100 ZM + 1.803 GS + 2.119 CG − 1.012 ZM.GS + 0.953 ZM.CG + 1.534 CG.GS					
b_1_	2.100	1.60	0.106	19.90	S (*p* ˂ 0.001)
b_2_	1.803	1.60	0.106	17.09	S (*p* ˂ 0.001)
b_3_	2.119	1.60	0.106	20.08	S (*p* ˂ 0.001)
b_12_	−1.012	1.57	0.485	−2.09	NS
b_13_	0.953	1.57	0.485	1.96	NS
b_23_	1.534	1.57	0.485	3.16	S (*p* ˂ 0.05)
Y_5_ = 66.816 ZM + 65.021 GS + 64.16 CG + 7.913 ZM.GS − 3.927 ZM.CG − 2.577 GS.CG					
b_1_	66.816	1.60	0.430	155.53	S (*p* ˂ 0.001)
b_2_	65.021	1.60	0.430	151.35	S (*p* ˂ 0.001)
b_3_	64.161	1.60	0.430	149.35	S (*p* ˂ 0.001)
b_12_	7.913	1.57	1.975	4.01	S (*p* ˂ 0.01)
b_13_	−3.927	1.57	1.975	−1.99	NS
b_23_	−2.577	1.57	1.975	−1.30	NS
Y_6_ = 2.234 ZM + 1.674 GS + 0.364 CG + 3.0502 ZM.GS − 2.170 ZM.CG − 1.210 GS.CG					
b_1_	2.234	1.60	0.022	102.14	S (*p* ˂ 0.001)
b_2_	1.674	1.60	0.022	76.53	S (*p* ˂ 0.001)
b_3_	0.364	1.60	0.022	16.62	S (*p* ˂ 0.001)
b_12_	3.050	1.57	0.101	30.34	S (*p* ˂ 0.001)
b_13_	−2.170	1.57	0.101	−21.59	S (*p* ˂ 0.001)
b_23_	−1.210	1.57	0.101	−12.04	S (*p* ˂ 0.001)
Y_7_ = 16.635 ZM + 18.215 GS + 31.670 CG − 0.268 ZM.GS + 12.142 ZM.CG + 4.842 GS.CG					
b_1_	16.635	1.60	0.158	104.99	S (*p* ˂ 0.001)
b_2_	18.215	1.60	0.158	114.96	S (*p* ˂ 0.001)
b_3_	31.670	1.60	0.158	199.87	S (*p* ˂ 0.001)
b_12_	−0.268	1.57	0.728	−0.37	NS
b_13_	12.142	1.57	0.728	16.67	S (*p* ˂ 0.001
b_23_	4.842	1.57	0.728	6.65	S (*p* ˂ 0.001)

GS: *Gymnosporia senegalensis*; ZM: *Ziziphus mauritiana*; CG: *Combretum glutinosum*; BS: *Boscia senegalensis*; CC: *Citrullus colocynthis*; S (*p* ˂ 0.001): significant at the level 99.9%; S (*p* ˂ 0.01): significant at the level 99%; S (*p* ˂ 0.05): significant at the level 95%; NS: nonsignificant.

## Data Availability

The original contributions presented in this study are included in the article. Further inquiries can be directed to the corresponding authors.
